# Frozen-Phase High-Pressure Destruction Kinetics of *Escherichia coli* as Influenced by Application Mode, Substrate, and Enrichment Medium

**DOI:** 10.3390/foods11121801

**Published:** 2022-06-18

**Authors:** Chunfang Wang, Hongru Liu, Yong Yu, Yongjin Qiao

**Affiliations:** 1 Crop Breeding & Cultivation Research Institute, Shanghai Academy of Agricultural Sciences, 1000 Jinqi Road, Fengxian District, Shanghai 201403, China; fhwcf@126.com (C.W.); hear2008dream@163.com (H.L.); 2 College of Biosystems Engineering and Food Science, Zhejiang University, 866 Yuhangtang Road, Hangzhou 310058, China; yyuzju@zju.edu.cn

**Keywords:** high pressure, frozen phase, BHIA medium, pulse mode, kinetics

## Abstract

The synergistic effect of frozen-phase high pressure (HP) on the inactivation of *E. coli* ATCC 25922 cultures in suspension medium, Chinese bayberry juice (pH 3.0), and carrot juice (pH 6.5) was evaluated. The survivor count of *E. coli* remained at 3.36 log CFU/mL on a nonselective brain heart infusion (BHIA) medium, while no survivor was detected on a selective violet red bile agar (VRBA) medium after a 5 min hold pressure at 250 MPa in a frozen culture suspension. BHIA was suitable for safe testing of the injured *E coli* cells after HP treatment in frozen state. Frozen Chinese bayberry juice showed higher sensitivity to HP treatment for its matrix property with high sterilizing efficiency at 170 MPa. Two pulses exhibited a significant inactivation effect in frozen samples compared with one pulse, especially for the Chinese bayberry juice with different pressure levels. The destruction kinetics of HP pulse mode followed the first-order rate kinetics with a *Z*p value of 267 MPa in frozen carrot juice. Our results evaluated the influenced factors of frozen HP destruction effects, including the medium, substrate, and application mode. The frozen HP destruction kinetics of pulses afford us better understanding of the technology application in the food industry.

## 1. Introduction

In the past decades, HP processing received extensive attention as an alternative to thermal processing, improving food safety and quality with extended food shelf life [1,2,3]. To lower the necessary peak pressure inactivating microorganisms in food, a combination of heat and pressure minimizes the treatment severity conditions, and exploring alternate/unconventional ways is the most researched aspect recently. A number of studies have revealed the synergistic effect of pressure and temperature to inactivate microorganisms [4,5,6]. However, research on subzero temperature (mainly targeting vegetative bacteria) has been far behind that on moderate and elevated temperatures. Shen et al. [7] reported that a 20 s treatment at 250 and 350 MPa at −25 °C in frozen *Bacillus subtilis* cell suspensions resulted in more than a 4 log reduction of CFU/mL in ACES (pH 8.5). Ponce et al. [8] found that HP inactivation was less pronounced at low temperatures (2 and −15 °C) than at room and higher temperatures in *Salmonella enteritidis*. However, later reports showed that the highest cell lethality in *Zygosaccharomyces bailii* and *Listeria monocytogenes* at 300 MPa was achieved at either 45 or −5 °C, and the lowest cell lethality was achieved at room temperature [6]. Thus, the thermodynamic state of water was crucial for the microbial destruction effects. Recently, a good synergistic effect on the sterilization of pressure and subzero temperatures was achieved in a special container reported in our previous studies [9,10]. Freezing prior to HP processing achieved better inactivation effect compared with unfrozen samples [11,12]. A 5.63 log reduction was obtained in samples frozen at −24 °C, while only a 1.83 log reduction was obtained in unfrozen samples at 4 °C following a 9 min treatment at 400 MPa [11]. Further studies are necessary to better understand the influenced factors of the inactivation effect of HP treatment, including frozen phase, pressure levels, application modes, substrates, and recovery medium.

In addition to the combination of temperature and pressure in HP, the application mode of pressure with multiple cycles or pulses was studied intensively. Some studies reported that multiple cycles or pluses enhanced lethality [4,13,14,15,16], while others found no benefit from pulse pressure application [17,18]. Donsì et al. [19] indicated that the effects of pressure pulse depended on combinations of pulse number, pressure level, and temperature. Although effects on the inactivation of microorganisms of multiple pulse HP treatments have been studied widely, the crucial factors remain unclear. Li et al. [10] found that two pressure pulses resulted in a higher lethal effect in frozen milk than in unfrozen milk. For instance, effects on frozen or subzero temperature levels are reported rarely.

Some studies reported that high-pressure treatment caused sublethal effect on some bacteria, and they regained viability in an appropriate environment [20,21]. To avoid the growth of survived cells and the recovery of injured ones during storage, especially at the appropriate temperature, it is necessary to determine the status of bacteria after HP treatment. Some reports have been aware of these problems [4,22]. A high injury level up to 55.98% in *Listeria monocytogenes* was induced in a frozen BHI broth (−80 °C), while it was 3.26% for samples without freezing (20 °C) [12]. The nonselective BHIA medium and the selective VRBA medium were used to enumerate the survived *E. coli* cells. BHIA is helpful for the recovery and growth of both normal and injured cells. Meanwhile, VRBA is a more selective medium for *E. coli* cells, which is rather harsh and does not allow the injured cells to grow. Then the differences between BHIA and VRBA counts represent those of the injured *E. coli* cells [4,10].

A natural food composed of a complex matrix with fats, proteins, mineral substances, and sugars influences the microbial resistance to HP [23,24]. Hence, it is desirable to simulate a natural food matrix to investigate the microbial destruction effect. The pH of a food matrix determines the inactivation kinetics extensively. A pH of 4.5 is a standard border of low-acid and high-acid foods. The low pH in high-acid food inhibits the germination and growth of spores. Carrot juice has previously been used in the static hold HP kinetics determination as a low-acid food matrix (pH 6.5), but has not been evaluated under pulsed HP applications [9]. Chinese bayberry (*Myrica rubra* Sieb. et Zucc.) is native to China with high nutrition and commercial value [25]. Chinese bayberry juice has been used as a candidate for the quality evaluation of HP treatment as a high-acid food matrix (pH 3.0) in our previous report [3].

In summary, several factors in HP treatment determined the final sterilizing effect, including frozen state, application mode, pH of the food matrix, and recovery medium in safe testing. A special equipment designed in our previous studies could fulfill frozen HP treatment conveniently and efficiently [9,26]. We have made progress in evaluating the effects of phase transitions, frozen state, and pressure on inactivating efficiency on *E. coli* in carrot juice [9]. Meanwhile, the destruction effect of the application mode and recovery medium on the inactivation of *E. coli* in a natural food matrix remains unclear. Chinese bayberry juice and carrot juice are widely accepted by consumers with their delicious flavor and high nutrition. Besides, Chinese bayberry juice and carrot juice represent high-acid and low-acid natural foods, respectively, with different pH’s. Therefore, the two juices are good materials for investigating the synergistic effect of factors in HP treatment. Above all, we evaluated and compared the HP destruction effect on *E. coli* systematically with considerations of states (frozen vs. unfrozen), application mode (static hold vs. pulse), substrate (pH dependent—culture suspension, carrot juice, Chinese bayberry juice) and recovery medium (BHIA vs. VRBA).

## 2. Materials and Methods

### 2.1. E. coli Culture Preparation

The culture of *E. coli* ATCC 25922 (CGMCC 1.2385) was obtained from the China General Microbiological Culture Collection Center (CGMCC, Beijing, China). Fresh *E. coli* culture was prepared with the same procedure as before [9] and cultured in a 50 mL sterile nutrient broth (Sinopharm Chemical Reagent Co., Ltd., Shanghai, China) twice with incubation at 37 °C for 24 h each. The initial population of *E. coli* was approximately 10^8^ colony forming units (CFU)/mL. Cell pellets were centrifuged from a 30 mL incubated *E. coli* broth at 3200× *g* for 5 min at 20 °C (5810R, Eppendorf AG, Hamburg, Germany) and stored in a refrigerator to be used to inoculate subsequent juice samples.

### 2.2. Sample Preparation

Carrot juice was prepared in a previous study and stored in a freezer (BC/BD-103HA, Haier, China) below −20 °C before use [9]. Fresh Chinese bayberry (*Dongkui*) was purchased from a local fruit market (Hangzhou, China). Whole Chinese bayberries were squeezed with a juice extractor (JYZ-B550, Joyoung Co., Ltd., Hangzhou, China), and the juice was then centrifuged at 3500× *g* for 10 min. The supernatants were filtered through a 0.23 mm pore diameter filter and then subjected to a pasteurization treatment in a thermostatically controlled water bath (DKS-224, Zhongxin Medical Instrument Co., Ltd., Jiaxing, China) at 65 °C for 30 min in order to kill the natural microflora. Finally, Chinese bayberry juice was aseptically packed into sterile bags (110 mm × 185 mm, BagLight PolySilk, Interscience, Saint Nom la Bretêche, France) (100 mL for each bag), sealed using a heat sealer (FS-300, Yongkang Teli Packing Machinery Co., Ltd., Jinhua, China), and stored in a freezer until use. Microbial enumeration of both stored juice samples was carried out before use on BHIA and resulted in negative counts. The clear carrot juice had a pH of 6.49 ± 0.12 (FE 20, Mettler Toledo, Shanghai, China), while the Chinese bayberry juice was 2.95 ± 0.03. Juice samples were thawed for 4 h at 4 °C prior to inoculation of *E. coli* culture.

A cultured nutrient broth was used as *E. coli* cell suspensions. As for Chinese bayberry and carrot juice, previously prepared *E. coli* pellets were separately resuspended in 20 mL thawed Chinese bayberry and carrot juice. Then they were added back into the thawed juice in a sterilized beaker to make an approximately 100 mL juice sample. After stirring at 500 rpm (RCT BS25, IKA, Staufen, Germany) for 1 min, the inoculated juice samples were aseptically transferred into sterile 2.0 mL cryogenic vials (430659, Corning Inc., New York, NY, USA). Then they were individually vacuum-packed with two layers of polyethylene bags. For HP treatment with frozen samples, the cryogenic vials were loaded into a specially designed plastic container. A detailed description is available in a previous report [9]. The containers with samples were filled with water and frozen at −20 °C overnight (about 16–18 h). Such a procedure made it possible to carry out the destruction kinetics with the sample still in the frozen state with the bulk of surrounding the test samples providing the bulk heat sink to prevent the test sample from thawing.

Samples frozen and stored at −20 °C without HP treatment were used as control of frozen samples. As for the HP treatment of unfrozen samples, packed sample vials were stored at 4 °C for control and subsequent HP treatment within 4 h.

### 2.3. HP Treatment

HP treatment was carried out using an experimental HP apparatus (UHPF-750, Baotou Kefa High Pressure Technology Co., Ltd., Baotou, China) with a maximum chamber capacity of 5 L. The pressure inside the HP vessel was detected by a pressure transmitter (HP-2, WIKA Alexander Wiegand SE & Co. KG., Klingenberg, Germany). A high-pressure unit was connected to a data logger (34970A, Agilent Technologies GMBH, Boblingen, Germany) for temperature recoding during HP treatment, similar to that in a previous report [27]. The pressure-transmitting medium was water, and the pressure vessel was maintained at room temperature (below 25 °C). Such a process can be replicated in other commercial HP equipment using the near-room-temperature setup, when specially designed containers, like the one described here [9], are used for application. The pressure come-up rate was about 150 MPa/min, and the depressurization time was less than 10 s. Data on pressure and temperature were recorded at a 1 s interval. For unfrozen samples, the temperature was kept below 33 °C.

Samples were subjected to HP treatment at selected pressure levels (170, 210, 250, 290, or 330 MPa) for one or multiple pulses (pressurization followed by a very brief static period of <5 s to reduce the strain on the equipment, followed by depressurization), respectively, considered to be including only pressurization and depressurization, thereby constituting a pulse pressure. A static hold HP treatment cycle with 5 min holding time (not including the pressure come-up and pressure release time) was also included to investigate the pressure holding time effect. After HP treatment, frozen samples carried by the plastic container were immersed in running cold water (room conditions) for thawing. They were checked periodically to ensure thawing (<4 °C) and avoid overthawing (>4 °C). Treated sample vials (thawed frozen vials and unfrozen vials) were held at 4 °C before microbial enumeration within 4 h.

### 2.4. Enumeration

Sample vials were opened and respectively transferred to a sterile tube, followed by serial 10-fold dilution in 0.1% peptone water (Sinopharm Chemical Reagent Co., Ltd., China). Surviving *E. coli* cells were enumerated independently on both BHIA (Hangzhou Tianhe Microorganism Reagent Co., Ltd., Hangzhou, China) and VRBA (Sinopharm Chemical Reagent Co., Ltd., Shanghai, China) using the pour plate method. For *E. coli* suspension and Chinese bayberry juice samples, both the BHIA and VRBA plate mediums were used, while BHIA plates were used for carrot juice samples. Survived *E. coli* colonies were enumerated after aerobic incubation at 37 °C for 48 h. Initial counts of *E. coli* in different sample suspensions were obtained likewise from control samples intended for both 4 °C (unfrozen samples) and −20 °C (frozen samples) applications.

### 2.5. Kinetic Analysis

The first-order kinetic model (Equation (1)) was used for pulse HP destruction kinetic analysis in this study, similar to that in Shao et al. [28]. *N_D_* was used as the number of pressure pulses required for achieving one decimal reduction in microbial population (thus equivalent to a *D* value) and was obtained from the regression of log (*N*t/*N*_0_) versus the number of pulses. Since pulse treatment can only be performed with a whole cycle, pulse effect (*PE*) was used as defining the log reduction can be achieved by one pulse HP treatment:

(1) $$\log\left(\frac{N_{t}}{N_{0}}\right)=-\frac{t}{N_{D}}$$

(2) $$N_{D}=-1/\left(slope\right)$$

(3) $$N_{D}=1/PE$$

where *N*_t_ is the number of *E. coli* survivors (CFU/mL) after different pulses of HP treatment, t is the number of pulses, and *N*_0_ is the initial number of *E. coli* (CFU/mL) in frozen samples at −20 °C without pulse HP treatment.

*Z*p value represents the required pressure for achieving one decimal reduction in *N_D_* values. It was determined as the negative reciprocal of the regression *slope* of plotting the decimal logarithm of *N_D_*-values log (*N_D_*) versus pressure:

(4) $$Zp=\left(P_{2}-P_{1}\right)/{\left(\log N_{D1}-\log N_{D2}\right)}_{}$$

*Z*p value characterized the pressure sensitivity of *E. coli* to pulse-mode HP treatment.

### 2.6. Statistical Analysis

Statistical analysis was performed using one-way analysis of variance (ANOVA). The Tukey test (*p* < 0.05) was applied to compare the differences of different HP destruction effects in average values by using SPSS 20.0 (Statistical Package for the Social Sciences, SPSS Inc., Chicago, IL, USA).

## 3. Results and Discussion

### 3.1. Temperature of Frozen Samples at Different Pressures

**Figure 1:**
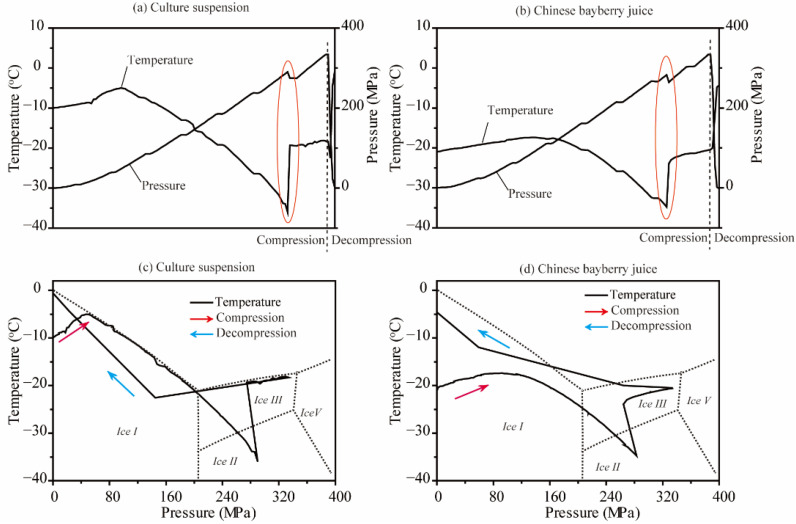
Changes in temperature and pressure of frozen culture suspension and Chinese bayberry juice samples of HP treatment at 330 MPa (**a**,**b**) and temperature–pressure profiles superimposed on the phase diagram of water (**c**,**d**).

The temperature change under different pressures during HP treatment with one pulse showed us the state transformation of both the culture suspension (Figure 1a,c) and Chinese bayberry juice (Figure 1b,d) clearly. As the pressure increased during the compression process, we can see that the metastable ice I region was positioned around 210–290 MPa in *E. coli* culture suspension. Then a sudden increase in the sample temperature (−36 to −19 °C) occurred (circled with a red line) (Figure 1a). Correspondingly, the pressure decreased suddenly from 289 to 275 MPa, which was a reasonable decline for the decrease in volume with intensity increase in the culture suspension from ice I (0.92 g/cm^3^) to ice III (1.14 g/cm^3^). Not surprisingly, the phase transition point of Chinese bayberry juice was different with the culture suspension with a sudden pressure change from 283 to 264 MPa and a temperature change from −35 to −24 °C (Figure 1b,d). The process was the classical phase transition with the exothermic reaction of the support medium under different pressures of HP treatment. The classic transformation relationship of the sample pressure and temperature indicated to us the phase transition of the support medium during HP treatment with specific pressures in one pulse. The state change and different phase transition point of the support medium have been reported in many studies, including those on carrot juice, milk, grape juice, apple juice [9,10,29,30]. The different phase point in a different support matrix implied the difference of inactivation efficiency with HP treatment, and the low pH matrix and frozen temperature were helpful to improve the inactivation efficiency in many reports [26,31]. The temperature change was important factors of the inactivation efficiency. Thus, we determined the temperature of the samples under different pressure levels with the same method (Table 1). All the temperatures were subzero and changed slightly, which kept all the three kinds of frozen samples in frozen status under all treatments. We selected different suitable pressure levels for the three different matrices according to the property and our previous reports [9,26].

### 3.2. High-Pressure Inactivation Effect on E. coli in Culture Suspension

**Figure 2:**
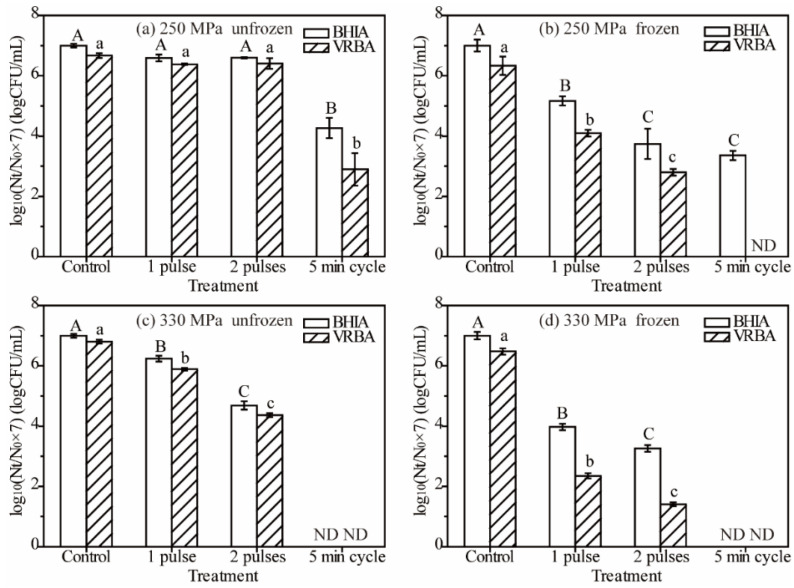
The *E. coli* survivors on both BHIA and VRBA plates in unfrozen and frozen culture suspensions after different HP treatments (250 MPa ((**a**) unfrozen, (**b**) frozen), 330 MPa ((**c**) unfrozen, (**d**) frozen)) and different pulses. Different letters (A, B, C, and D or a, b, c, and d) indicate a significant difference (*p* < 0.05) (when the same letter shares—not significant *p* > 0.05) between treatments on the same enumeration medium. ND means not detected. Mean values ± standard error (*n* = 3).

Survivors of *E. coli* were determined after HP treatments in culture suspension with different combinations of application modes, sample state, and pressure levels using the BHIA and VRBA mediums (Figure 2). The initial concentration of *E. coli* in different suspensions varied from 6.4 to 8.6 log CFU/mL due to different food matrices and temperatures. They were normalized to 10^7^ CFU/mL (survivors after each treatment divided by the initial count of BHIA and then multiplied by 10^7^) to graphically compare them easily among different treatments.

The survived *E. coli* cells in unfrozen samples after HP treatment of 250 MPa with one or two pulses had an insignificant difference compared with control, while they were significantly reduced at 330 MPa HP treatment (Figure 2a,c). Survivors of an unfrozen *E. coli* sample after 250 MPa with a 5 min cycle treatment were 4.26 log CFU/mL on BHIA count, but they reduced to a undetectable level at 330 MPa with the same 5 min cycle (Figure 2a,c). They showed a similar trend for frozen samples, and the VRBA medium survived few cell even at 250 MPa under the 5 min cycle (Figure 2b,d). An application mode of the 5 min cycle inactivated the *E. coli* cells more effectively than two pulses in unfrozen samples at both 250 and 330 MPa (Figure 2a,c). Meanwhile, the effect was not significant compared with two pulses in the frozen sample with the BHIA medium at 250 MPa (Figure 2b). The inactivated effect of the two-pulse HP treatment was significantly higher than that of the one-pulse treatment except for the unfrozen samples at 250 MPa (Figure 2). These results indicated that multiple pulses inactivated the *E. coli* cells more effectively, especially in combination with higher pressure and frozen state. Pilavtepe-Çelik et al. [22] reported that an increased pulse number of HP treatment with or without holding time at 300 MPa in peptone water at ambient temperature increased the inactivation effect. In addition, HP treatment in unfrozen apple juice at 350 MPa showed that one cycle with 5 min holding could achieve a similar inactivation effect compared with a four-pulse treatment [4].

Frozen status enhanced the inactivation effects of HP treatment on *E. coli* obviously in both multiple pulses and 5 min cycle application modes (Figure 2). For instance, the two pulses showed no significant effects on the inactivation of *E. coli* cells in an unfrozen sample compared with control (Figure 2a). However, the survived *E. coli* cells reduced significantly with 1.83 and 3.26 log CFU/mL reductions in one and two pulses, respectively, compared with control in a frozen sample at 250 MPa (Figure 2b). The results indicated that the frozen state showed a good synergetic effect with high pressure on *E. coli* inactivation, which was consistent with our previous studies [9,26].

Interestingly, the remaining cell was undetectable with VRBA in frozen samples with the 5 min cycle at 250 MPa. However, we still detected the survived cells in a nonselective BHIA medium with a count of 3.36 log CFU/mL (Figure 2b). These results indicated that the BHIA medium was more suitable for safe testing after HP treatment at 250 MPa in frozen culture suspensions.

Notably, the BHIA medium could rejuvenate and recover the injured cells, while the VRBA medium could not for its stronger selective stress for injured *E. coli* cells. Thus, the different survived *E. coli* cells in a frozen sample at 250 MPa after the 5 min cycle indicated the existence of injured cells after HP treatment at these conditions. In addition, the existence of injured cells after HP treatment have been reported in previous studies, and the pressure treatment at 193 MPa at −20 °C remained the recovery of the injured cells [32]. Pilavtepe-Çelik et al. [22] stated that a selective medium yielded higher inactivation results than a nonselective medium of *E. coli* regardless of the pulse numbers and holding time. The results indicated that using a selective medium (VRBA) for the detection of *E. coli* may overestimate the reduction even at subzero temperatures with high pressure, unless the pressure treatment is severe enough to destroy the microbial population completely. It is necessary not only to challenge the HP process with microbial cells in order to establish the process but also to check for the possibility of the presence of injured cells, especially when food was involved, since they could revive and grow under appropriate growth conditions. Therefore, using a nonselective medium (such as BHIA) is a better option; however, this medium cannot be used when mixed cultures are present because it is not selective for *E. coli*.

### 3.3. High-Pressure Inactivation Effect on E. coli in Chinese Bayberry Juice

**Figure 3:**
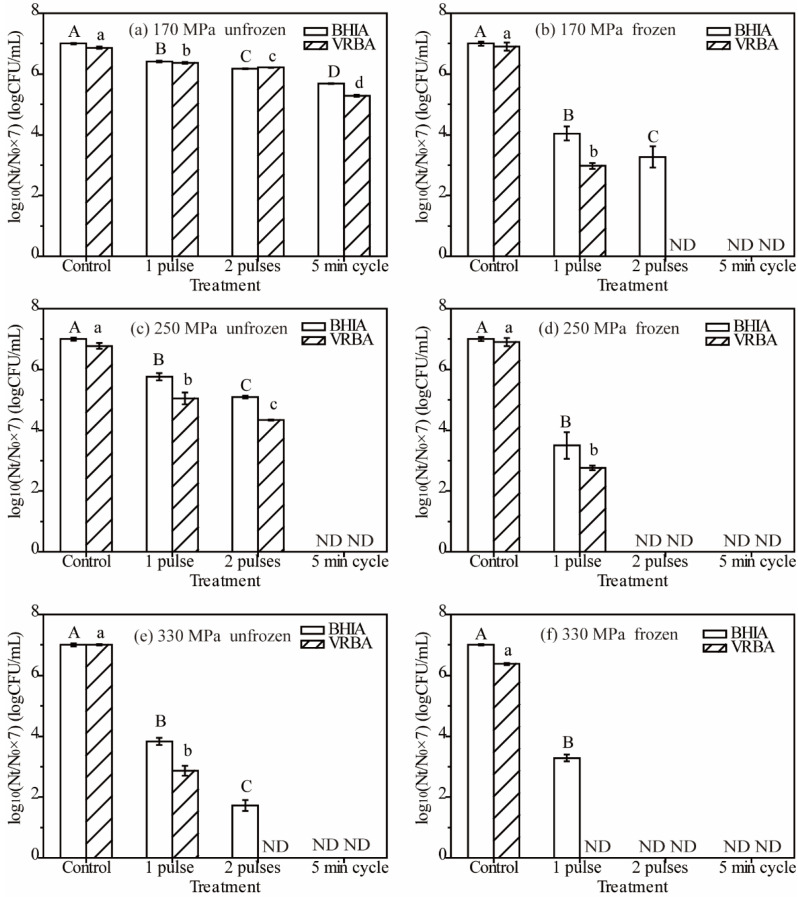
The *E. coli* survivors in unfrozen and frozen Chinese bayberry juice after different HP treatments (170 MPa ((**a**) unfrozen, (**b**) frozen), 250 MPa ((**c**) unfrozen, (**d**) frozen), 330 MPa ((**e**) unfrozen, (**f**) frozen)) on BHIA and VRBA. Different letters (A, B, C, and D or a, b, c, and d) indicate a significant difference (*p* < 0.05) (when the same letter shares—not significant *p* > 0.05) between treatments on the same enumeration medium. ND means not detected. Mean values ± standard error (*n* = 3).

Since Chinese bayberry juice belongs to the category of high-acid natural food with pH = 3.0, a lower pressure level of 170 MPa was used to determine the inactivated effects, compared with the other two higher pressure levels (Figure 3). The survived *E. coli* cells reduced significantly in both frozen and unfrozen samples with two mediums after 170 MPa HP treatments with either multiple pulses or the 5 min cycle (Figure 3a,b). The higher destruction efficiency might be due to the high-acid property of Chinese bayberry juice. Although the survived *E. coli* cells reduced significantly after 170 MPa HP treatment of two pulses in unfrozen Chinese bayberry juice, the amplitude of variation was small (Figure 3a). However, a much higher reduction of survivors was obtained with frozen samples in two pulses with the higher pressure levels (Figure 3c–f). As shown in Figure 3b, *E. coli* cells were undetected after 5 min cycle treatment at 170 MPa in frozen samples. The synergetic effects on the inactivation of *E. coli* cells of freezing and pressure were consistent with the results in culture suspension (Figure 2).

The advantage of a nonselective BHIA medium in safe testing after HP treatments was obvious, and the survived injured cells were covered up by the selective VRBA medium in frozen Chinese bayberry juice under two pulses at 170 MPa and even one pulse at 330 MPa (Figure 3b,f). Further, the unfrozen sample also exhibited the phenomena under two pulses at 330 MPa (Figure 3e). Thus, the selection of the medium is important and specific with different food matrices, for the probability of ignoring survived injured cells in Chinese bayberry juice is higher than that in culture suspension (Figure 2 and Figure 3). Considering the complexity of the natural food matrix, the ignored survived injured cells after HP treatment were a potential risk in food safety and quality because several reports have indicated the existence of injured cells after HP treatment [10,12,33]. The injured cell might revive and grow during storage, spoiling the food and causing risk. For instance, sublethal injured cells were detected in tomato juice (pH 4.1) during low-temperature storage even after HP treatment of 450 MPa with both *E. coli* O157 and *Listeria monocytogenes* with the MacConkey and TSAUP mediums [34]. The regeneration of sublethal injured cells suspended in carrot juice was observed during storage after HP treatment, and the level of injured *E. coli* cells about doubled (5.43 log CFU/mL) after 28 days’ storage [35].

In addition, the intensity of pressure was a significant factor of sterilizing efficiency in *E. coli* cells in either unfrozen or frozen samples in both multiple pulses and the 5 min cycle (Figure 3). For instance, the counts of *E. coli* cells reduced by nearly 5 log CFU/mL at 330 MPa in the BHIA medium compared with 170 MPa in unfrozen samples (Figure 3a,e). The survived cell was undetected when pressure exceeded 250 MPa with the application of the 5 min cycle in unfrozen samples (Figure 3c,e).

The results indicated that the number of pulses exhibited significant influence on the inactivation effect of HP treatment in either unfrozen or frozen samples with various pressure levels in Chinese bayberry juice.

### 3.4. Pressure Pulse Destruction Kinetics of E. coli in Frozen Carrot Juice

**Figure 4:**
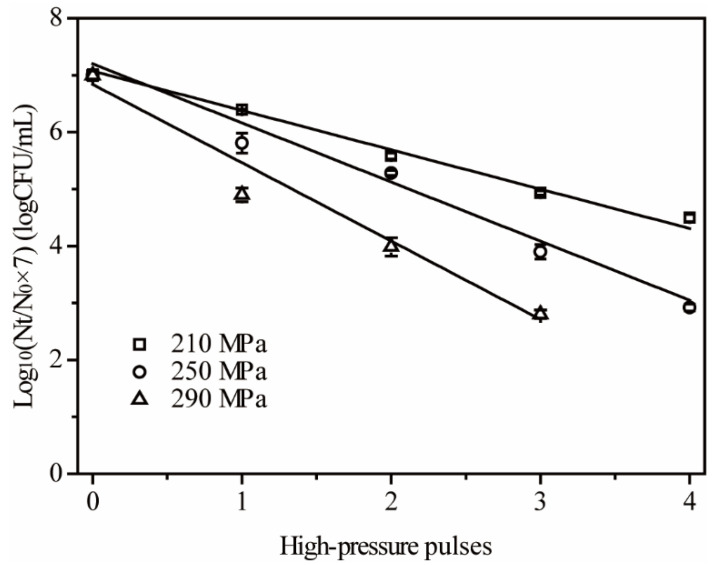
*E. coli* survivors in frozen carrot juice samples after multiple-pulse HP treatments.

The results in Section 3.2 and Section 3.3 indicated that the efficiency of multiple pulses depended on the frozen state and pressure levels of the samples. Therefore, we studied the pressure pulse destruction kinetics of *E. coli* in frozen carrot juice (Figure 4 and Table 2). The logarithmic survivor curves of *E. coli* in frozen carrot juice under three different pressure levels were used to analyze the destruction kinetics of HP pulse treatment. The analyzed curves showed us that the slope increased as the pressure levels elevated, indicating the increased sterilizing efficiency, and the survived cells were undetected when the pulses reached four at 290 MPa (Figure 4). The relationship between the number of pressure pulses and the count of survivors all followed the first-order rate kinetic with three different pressure levels (*R*^2^ > 0.97) (Table 2). A reduction of 1 log CFU/mL at 210 MPa needed a 1.45 pulse process, and the fulfillment of the goal needed two pulses in practical application. When the pressure reached 250 MPa, we only needed 0.96 pulse to reduce 1 log CFU/mL, and the theoretical pulse decreased to 0.73 at 290 MPa (Table 2). Correspondingly, one pulse treatment achieved a 0.69 log CFU/mL reduction at 210 MPa, and it achieved a 1.03 log CFU/mL reduction at 250 MPa (Table 2). Thus, a pressure of 250 MPa needed five pulses to get a 5 log CFU/mL reduction, and 290 MPa needed four pulses. Considering the complexity of natural food and safety, 290 MPa might be suitable for practical use.

The pulse mode only included the pressurization and decompression processes, and four pulses needed about 7 min (1.5 min for each pulse and approximately 1 min depressurization process) at 290 MPa. However, we needed 13 min holding pressure plus a 1.5 min pressurization process to achieve a 5 log CFU/mL reduction at the same pressure level in frozen carrot juice in our previous report [9]. In summary, the HP pulse mode combined with the frozen state exhibited a potential to reduce the processing time compared with the static holding mode and elevated the efficiency of HP treatment. The efficiency comparison between pressure pulse and pressure hold mode was not consistent for various factors. For example, Ramaswamy et al. [4] reported a comparison of inactivation efficiency between multiple pulses and holding modes at 350 MPa with unfrozen apple juice. An 8 log-cycle reduction compared with an initial point needed a four-pulse process with a *PE* value of 2.17 log-cycle reduction. However, the holding mode of one pulse combined with a 5 min holding time could cause more than an 8 log-cycle reduction at 350 MPa. Considering the damage to the equipment of HP treatment, the pressure hold mode would be more desirable in practice. Donsì et al. [36] reported the effect of processing variables of HP treatment on the inactivation of *Saccharomyces cerevisiae* cells in fruit juices, including the pressure levels, number of process cycles, pulse holding time, and ramp rate. The results indicated that the pulse duration and number of pulses mainly determined the inactivation efficiency of HP treatment. The multiple-pulsed HP process with a threshold value of 10 cycles showed higher inactivation efficiency than the isostatic application mode in orange juice at both 150 and 200 MPa. The result was consistent with ours, where exhibiting a higher HP treatment effect could be achieved by a reasonable number of pulses compared with the isostatic state. In addition, a multiple-pulsed process could reduce the recovery of injured *E. coli* cells compared with a single-pulsed process in peptone water during cold storage after HP treatment [22]. The efficiency of pulsed HP processes depends on the combination of pulse holding time, number of pulses, food matrix, and bacterial difference, as well as ramp rate and process temperature. The results showed the synergistic effect of temperature and multiple pulses. Meanwhile, the pulse numbers exhibited enhanced effect with pressure levels in frozen Chinese bayberry juice and carrot juice.

The destruction kinetics of a frozen HP pulse mode have been reported rarely, though the influences of pulses, temperatures, pressure levels, and food matrix on the inactivation effect have been discussed a lot. The *Z*p value of the HP pulse mode was 267 MPa, which means the elevation of pressure needed for one decimal reduction in *N_D_* values. Meanwhile, the pressure elevation was 613 MPa in frozen carrot juice with the HP holding mode [9]. The result indicated that the pressure sensitivity of the HP pulse mode was higher than that of the HP holding mode, implying the high efficiency and flexible application in practice. In summary, the HP pulse destruction effect on *E. coli* followed the first-order rate kinetic well in frozen carrot juice. The kinetics explained the effect of multiple pulses on the inactivation of *E. coli* and afforded the foundation for other studies in various matrices.

## 4. Conclusions

HP treatment has been widely studied in various matrices and at an elevated temperature with different pressure levels; however, the frozen state has shown high efficiency on the inactivation of *E. coli*, and the influence factors were discussed in our work. The injured *E. coli* cells were observed in both unfrozen and frozen samples, which indicated the necessary use of BHIA in the safe testing of *E. coli* after HP treatment. The property of the matrix influenced the HP inactivation effect, causing the different efficiency of culture suspension, Chinese bayberry juice, and carrot juice. Two pulses showed better inactivation effects in neutral culture suspension than one pulse, and a remarkable elevation of efficiency was observed in frozen Chinese bayberry juice combined with higher pressure. Therefore, we further determined the destruction kinetics of HP multiple pulses in frozen carrot juice. The regression coefficient between the pressure and pulses was all higher than 0.97 and demonstrated that it followed the first-order rate kinetics well. The pulse mode in HP treatment showed high-pressure sensitivity with a 267 MPa *Z*p value, calculated from the first-order rate kinetics. In summary, the destruction effect on *E. coli* was affected by application modes, substrate, and recovery mediums in frozen status with various pressure levels. The kinetics of frozen high-pressure HP pulses afford us to help use the technology in the food industry.

## Figures and Tables

**Table 1:** Temperature–pressure conditions employed for the destruction of *E. coli* in culture suspension, Chinese bayberry juice, and carrot juice.

Samples\Temperature (°C)	Pressure (MPa)				
	170	210	250	290	330
Culture suspension	—	—	−28.3 ± 0.6	—	−18.7 ± 0.6
Chinese bayberry juice	−20.7 ± 0.4	—	−29.8 ± 0.3	—	−20.3 ± 0.5
Carrot juice	—	−21.7 ± 0.1	−26.8 ± 0.2	−18.6 ± 0.6	—

**Table 2:** Decimal reduction pressure pulse number (*N_D_*, mean ± SE) and pulse effect (*PE*, mean ± SE) on *E. coli* in frozen carrot juice.

Pressure (MPa)	*N_D_*	*PE* Value (log CFU/mL)	*R* ^2^_Adj_	*Z*p Value
210	1.45 ± 0.03 ^1^	0.69 ± 0.01	0.990	267 ± 28 MPa *R*^2^ = 0.978
250	0.96 ± 0.02	1.03 ± 0.02	0.978	
290	0.73 ± 0.02	1.37 ± 0.04	0.972	

^1^ SE was determined based on the SE of slope that resulted from the regression analysis of logarithm survivor versus pressure pulse number.

## Data Availability

Data is contained within the article.
